# Associations of load and progression of imaging markers of microvascular lesions and neurodegeneration with odor identification decline: a population‐based study

**DOI:** 10.1002/alz.086797

**Published:** 2025-01-09

**Authors:** Javier Oltra, Grégoria Kalpouzos, Ingrid Ekström, Maria Larsson, Chengxuan Qiu, Erika J Laukka

**Affiliations:** ^1^ Aging Research Center, Karolinska Institutet and Stockholm University, Stockholm Sweden; ^2^ Gösta Ekman Laboratory, Stockholm University, Stockholm Sweden; ^3^ Stockholm Gerontology Research Center, Stockholm Sweden

## Abstract

**Background:**

Olfactory deficits are predictive of cognitive decline and dementia. Previous studies have linked brain magnetic resonance imaging markers of neurodegeneration to olfactory deficits in aging; however, these studies analyzed cross‐sectional data for markers, olfaction, or both. Furthermore, potential cerebrovascular contributions to understanding why olfactory deficits predict dementia remain unexplored. We aimed to examine whether markers of microvascular lesions and neurodegeneration and their progression are associated with the rate of olfactory decline in community‐dwelling older adults.

**Method:**

Participants were 405 individuals (mean age=70.2 years; 60% females) from the Swedish National study on Aging and Care in Kungsholmen (SNAC‐K) who were free from dementia at baseline. We analyzed microvascular lesions (presence of lacunes, white matter hyperintensities volume, global perivascular spaces [PVS] count) and neurodegeneration (lateral ventricular, hippocampal, and total gray matter volumes) markers, available up to 6 years and an olfactory measure (Sniffin’ Sticks odor identification task), available up to 15 years. Additionally, we assessed summary scores for microvascular lesions and neurodegeneration load (range 0–3), accounting for all markers of each category (1 point for its presence or for being in the worst quartile, as appropriate). Data were analyzed with linear mixed‐effects models.

**Result:**

After adjustment for sociodemographic (age, sex, and education) and extra covariates (smoking status and semantic memory), a higher global PVS count (β =‐0.041; 95% CI, ‐0.077 to ‐0.005) and a high load (≥2 points) of neurodegeneration (β=‐0.112; 95% CI, ‐0.221 to ‐0.002) at baseline were associated with a faster olfactory decline. Longitudinally (n=228), faster accumulation of PVS (β=‐1.812; 95% CI, ‐3.474 to ‐0.150), faster hippocampal atrophy (β=1.348; 95% CI, 0.413 to 2.283), and an increment from low (≤1 point) to high load for microvascular lesions (β=‐0.167; 95% CI, ‐0.327 to ‐0.007) and neurodegeneration (β=‐0.206; 95% CI, ‐0.321 to ‐0.092) were associated with an accelerated olfactory decline.

**Conclusion:**

Both microvascular lesions and neurodegeneration are longitudinally associated with olfactory decline in aging. This relationship supports the notion that olfactory deficits could be valuable as an early marker for dementia. More longitudinal studies are needed to untangle the mechanisms in play between cerebral vascular burden, atrophy, and olfaction.

